# Differential regulation of H3S10 phosphorylation, mitosis progression and cell fate by Aurora Kinase B and C in mouse preimplantation embryos

**DOI:** 10.1007/s13238-017-0407-5

**Published:** 2017-04-22

**Authors:** Wenzhi Li, Peizhe Wang, Bingjie Zhang, Jing Zhang, Jia Ming, Wei Xie, Jie Na

**Affiliations:** 1 Center for Stem Cell Biology and Regenerative Medicine, School of Medicine, Tsinghua University, Beijing, 100084 China; 2 Center for Stem Cell Biology and Regenerative Medicine, MOE Key Laboratory of Bioinformatics, THU-PKU Center for Life Sciences, School of Life Sciences, Tsinghua University, Beijing, 100084 China

**Keywords:** Aurora kinase, mouse preimplantation embryo, cell fate, development, mitosis

## Abstract

Coordination of cell division and cell fate is crucial for the successful development of mammalian early embryos. Aurora kinases are evolutionarily conserved serine/threonine kinases and key regulators of mitosis. Aurora kinase B (AurkB) is ubiquitously expressed while Aurora kinase C (AurkC) is specifically expressed in gametes and preimplantation embryos. We found that increasing AurkC level in one blastomere of the 2-cell embryo accelerated cell division and decreasing AurkC level slowed down mitosis. Changing AurkB level had the opposite effect. The kinase domains of AurkB and AurkC were responsible for their different ability to phosphorylate Histone H3 Serine 10 (H3S10P) and regulate metaphase timing. Using an Oct4-photoactivatable GFP fusion protein (Oct4-paGFP) and fluorescence decay after photoactivation assay, we found that AurkB overexpression reduced Oct4 retention in the nucleus. Finally, we show that blastomeres with higher AurkC level elevated pluripotency gene expression, which were inclined to enter the inner cell mass lineage and subsequently contributed to the embryo proper. Collectively, our results are the first demonstration that the activity of mitotic kinases can influence cell fate decisions in mammalian preimplantation embryos and have important implications to assisted reproduction.

**Electronic supplementary material:**

The online version of this article (doi:10.1007/s13238-017-0407-5) contains supplementary material, which is available to authorized users.

## Introduction

Precise and timely segregation of sister chromatids during cell division is essential for early embryo development. In mammalian preimplantation embryos, chromosome segregation errors happen frequently, this can lead to deleterious downstream events, such as chromosome number and structure alteration, DNA damage, cytokinesis failure, which eventually lead to embryo arrest or severe developmental defects (Bolton et al., 2016; Vanneste et al., 2009; Wong et al., 2010). Global gene expression profiling studies discovered that many cell division kinases and regulators are highly enriched in preimplantation embryos (Xue et al., 2013). The high rate of cell division error implies that the regulation of mitosis, particularly the activity of cell division checkpoints, might be different from that in somatic cells. Spindle assembly checkpoint (SAC) is a surveillance mechanism safeguards the accurate separation of sister chromatids (Musacchio, 2015). It remains active until all kinetochores attach to spindle microtubules correctly (Musacchio, 2015). When SAC is active, Aurora kinase B (AurkB) phosphorylates and inactivates CDC20, to prevent the activation of anaphase-promoting complex/cyclosome (APC/C) (Hagting et al., 2002; Nasmyth, 2005; Ruchaud et al., 2007). Upon the inactivation of SAC, or reduction in AurkB activity, APC/C becomes active and ubiquitinates securin which leads to its degradation (Hagting et al., 2002). Separase, which is repressed by securin, becomes activated and cleaves the components of cohesin to permit the segregation of sister chromatids (Schvartzman et al., 2010). Thus, securin protein level negatively correlates with the activity of SAC, and the rate of its degradation reflects the dynamics of SAC inactivation during mitosis (Hagting et al., 2002). Due to the difficulties of manipulating the mouse preimplantation embryo, there has not been any study using securin degradation to study SAC activity in this system.

AurkC is a mammalian specific Aurora kinase closely related to AurkB (Carmena and Earnshaw, 2003; Lin et al., 2014; Sasai et al., 2004). It is specially highly expressed in mammalian gamete and required for spermatocyte meiosis (Dieterich et al., 2007). During oocytes meiosis I, AurkC displayed distinct function from AurkB (Balboula and Schindler, 2014; Sharif et al., 2010). It regulates localized chromosome passenger complex (CPC) activity and kinetochore microtubule (K-MT) attachments during the metaphase of meiosis I, thus promoting APC/C activation and securin degradation, while too much AurkC resulted in cytokinesis failure in meiotic I (Balboula and Schindler, 2014; Sharif et al., 2010). After fertilization, a significant number of AurkC^−/−^ embryos showed division defect which results in subfertility, while AurkB seemed not required in cleavage stage embryos but become important in blastocysts (Fernandez-Miranda et al., 2011; Schindler et al., 2012). In human, both AurkB and AurkC are expressed in preimplantation embryos, and AurkC level remained high until 8-cell stage (Avo Santos et al., 2011). Above studies highlighted the importance of AurkB and C function during preimplantation development, however, a direct comparative analysis of their mitotic roles in preimplantation embryos has been lacking and whether their activity may affect cell fate decision is another intriguing question.

In mammalian preimplantation embryos, cell division timing appeared to associate with cell fate choices (Chazaud and Yamanaka, 2016; Zernicka-Goetz, 2006). At 8-cell stage, the earlier dividing cells were more likely to locate to the inside of the morula, while the later dividing cells staying at outside (Tabansky et al., 2013; Zernicka-Goetz, 2006; Zernicka-Goetz et al., 2009). Subsequently, the inside cells gained advantage to enter the inner cell mass (ICM) lineage and outside cells become the trophecoderm (TE). Thus, the prior cell division advantage was accumulated through the first three cell divisions after fertilization (Zernicka-Goetz, 2006).

Here by using mRNA overexpression, siRNA knocking-down and live imaging, we show that the speed of mitosis and the degradation rate of securin in mouse preimplantation embryos can be controlled by the level of AurkB and AurkC. Further, AurkB and AurkC had different influence on the nuclear dynamics of Oct4 as shown by an Oct4-photoactivatable GFP fusion protein (Oct4-paGFP). Increasing AurkC level promoted pluripotency gene expression. Finally, we show that cells with more AurkC protein tend to locate to ICM and contribute to the embryo proper. Our results demonstrated that difference in the activity of cell division kinases has significant impact on cell fate decision.

## Results

### Aurora kinase B and C differentially regulate mitosis progression in mouse preimplantation embryos

**Figure 1:**
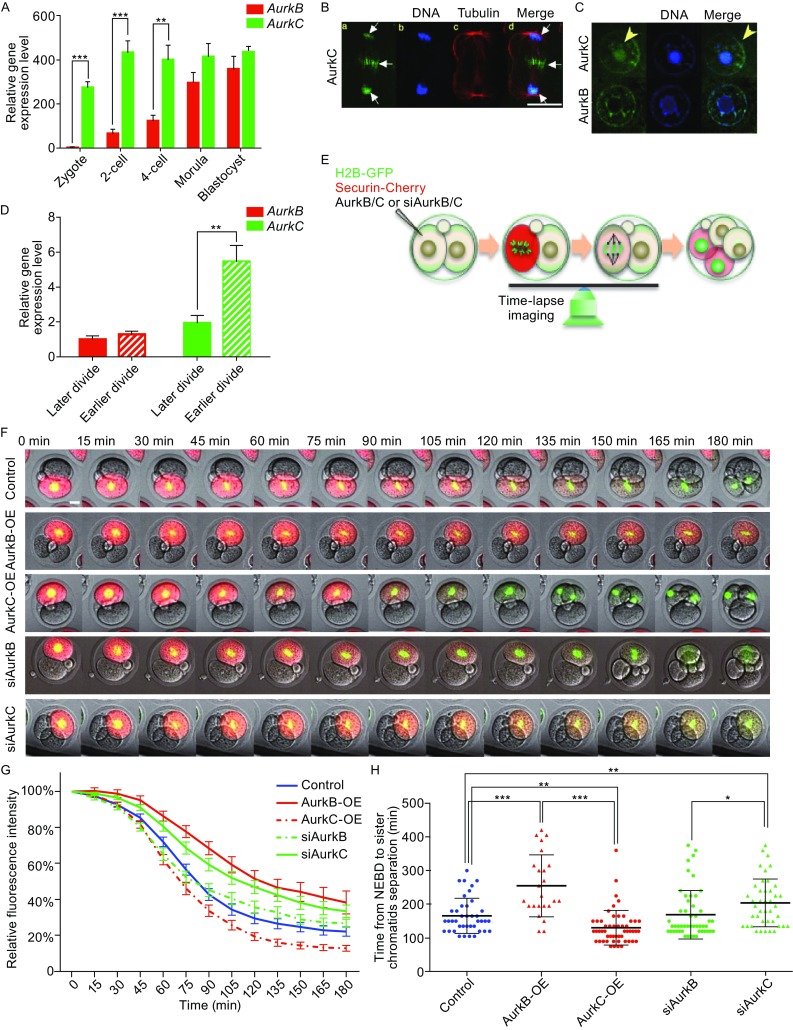
**AurkB and AurkC have different effect on Securin degradation and sister chromatids separation**. (A) Q-PCR analysis showing AurkB and AurkC mRNA expression level during mouse preimplantation development, each sample were normalized by AurkB level at zygote stage, the bar and whiskers indicate means and SEM, **P < 0.01, ***P < 0.001. (B) AurkC localization in a dividing blastomere. DNA (blue), AurkC (green), Tubulin (red). Scale bar, 40 μm. (C) The localization of AurkB and AurkC (green) during interphase, DNA (blue). Scale bars, 20 μm. (D) Q-PCR analysis showing AurkB and AurkC expression level in the daughter cells from earlier and later dividing cells, the level of AurkB in the later dividing cells was considered “1”, the bar and whiskers indicate means and SEM, **P < 0.01. (E) Schematic view of live imaging and Securin-mCherry degradation assay. (F) Selected frames from time lapse imaging movies of Securin-mCherry degradation in Control, AurkB-OE, AurkC-OE, siAurkB, and siAurkC groups embryos. Securin-mCherry (red), H2B-GFP (green), time interval, 15 min. Scale bars, 20 μm. (G) The Securin-mCherry degradation analysis in control (*n* = 40), AurkB-OE (*n* = 24), AurkC-OE (*n* = 52), siAurkB (*n* = 50), and siAurkC (*n* = 42) groups, from NEBD to chromosome separation. Y-axis, relative fluorescence intensity; X-axis, time in minutes (min). The continuous line and whiskers indicate means and SEM. (H) Quantification of the cell division time (from NEBD to chromatin separation, time/min, Y-axis) in control (n = 40), AurkB-OE (*n* = 24), AurkC-OE (*n* = 52), siAurkB (*n* = 50), and siAurkC (*n* = 42) groups (X-axis), each dot represents one embryo in the group, and the bar and whiskers indicate means and SD, *P < 0.05, **P < 0.01, ***P < 0.001

Previously, we showed that AurkB and AurkC differentially regulated securin degradation during mouse oocyte meiosis (Sharif et al., 2010). However, it is unclear whether this is also the case during embryo mitosis. We first examined AurkB and AurkC expression in mouse preimplantation embryos. Q-PCR analysis showed that AurkC was highly expressed throughout preimplantation stages, and AurkB was hardly detectable before 2-cell stage. From 2-cell to morula stage, although AurkB was transcribed, its level was still lower than that of AurkC (Fig. 1A). Next we used HA tagged AurkB and AurkC to reveal their subcellular distribution (Fig. 1B and 1C). During mitosis, AurkC associated with the chromosome, and translocated to the midbody between the two daughter cells during anaphase (Fig. 1B). During interphase, both AurkB and AurkC showed nuclear localization, while AurkC but not AurkB was seen in the nucleolus (Fig. 1C). During preimplantation experiment, the two blastomeres of the 2-cell embryo often divide asynchronously. We also compared AurkB and AurkC expression level in earlier and later dividing cells at early 4-cell stage. Mouse embryos were first cultured to very late 2-cell stage and then monitored every hour. Embryos with 3 cells (one blastomere has divided) were picked and dissociated. The daughter cells from the early and late dividing blastomere were separated and cultured to early 4-cell stage. Q-PCR analysis showed that earlier dividing cells had higher AurkC level but similar AurkB level compared with late dividing cells (Fig. 1D).

Next we asked whether cell division kinetics in preimplantation embryos can be altered by manipulating AurkB and AurkC levels. AurkB or AurkC mRNA or siRNA were co-injected with Securin-mCherry and H2B-GFP mRNAs into one blastomere of 2-cell embryos and time-lapse microscopy was used to monitor the speed of Securin degradation (Fig. 1E). The specificity of siRNA targeting AurkB and AurkC, and overexpression level of mRNAs were confirmed (Fig. S1B and S1C). During interphase, Securin-mCherry showed diffused cytoplasmic distribution and maintained a relatively stable level. The intensity of the red fluorescence represented Securin protein level, its value just before the nuclear envelope break down (NEBD) was considered as 100%. In the control blastomeres injected with Securin-mCherry and H2B-GFP mRNAs, 90 min after NEBD, Securin level decreased to 50% of the initial level. After 180 min, when the red fluorescence reached about 20%–30% of the initial level, the sister chromatids separated (Fig. 1F and Movie S1A–E). Overexpression of AurkB (AurkB-OE) significantly increased the time required for Securin-mCherry degradation. It took almost 150 min for Securin to reduce to 50%, and after 180 min, Securin was still at 40% level (Fig. 1G). During the prolonged metaphase, the chromosomes remain condensed (Fig. 1F), while sister chromatids stayed at the equatorial plan of the cell and failed to separate (Fig. 1F). On the other hand, overexpression of AurkC (AurkC-OE) significantly accelerated Securin-mCherry degradation, the red fluorescence reduced 50% within 75 min (Fig. 1G). Moreover, it reached less than 20%, even lower level than in control cells (Fig. 1G). Conversely, knocking down of AurkC through siRNA (siAurkC) in blastomeres delayed 50% Securin-mCherry degradation time to 120 min (Fig. 1G), similar to the timing in AurkB overexpression blastomeres. Sister chromatid separation was also delayed in AurkC knock-down cells (Fig. 1G). AurkB knock-down (siAurkB) cells had similar 50% Securin-degradation time as control cells. Consequently, the time required to complete cell division was also affected by altering AurkB and AurkC levels (Fig. 1H). The control group and siAurkB group had similar cell division time, 166.1 ± 8.240 min (*n* = 40) and 169.2 ± 10.16 min (*n* = 50) respectively. The cell division time was the longest in AurkB-OE group, 255.0 ± 17.96 min (*n* = 25). SiAurkC group had the second longest division time, 204.3 ± 10.93 min (*n* = 42). AurkC-OE group had the shortest division time, 130.8 ± 6.838 min (*n* = 54). Consequently, AurkC-OE cells tend to divide earlier than sister uninjected cells, and AurkB-OE and siAurkC cells divided later than uninjected cells (Fig. S1D). The above results suggested that in cleavage stage embryos, the timing of division can be controlled by varying AurkB and AurkC levels. AurkC appeared to be the major Aurora kinase regulating the mitosis speed, while too much AurkB seemed not compatible with the fast cell cycle progression rate in preimplantation development.

### The kinase domains of AurkB and C were responsible for their distinct functions

**Figure 2:**
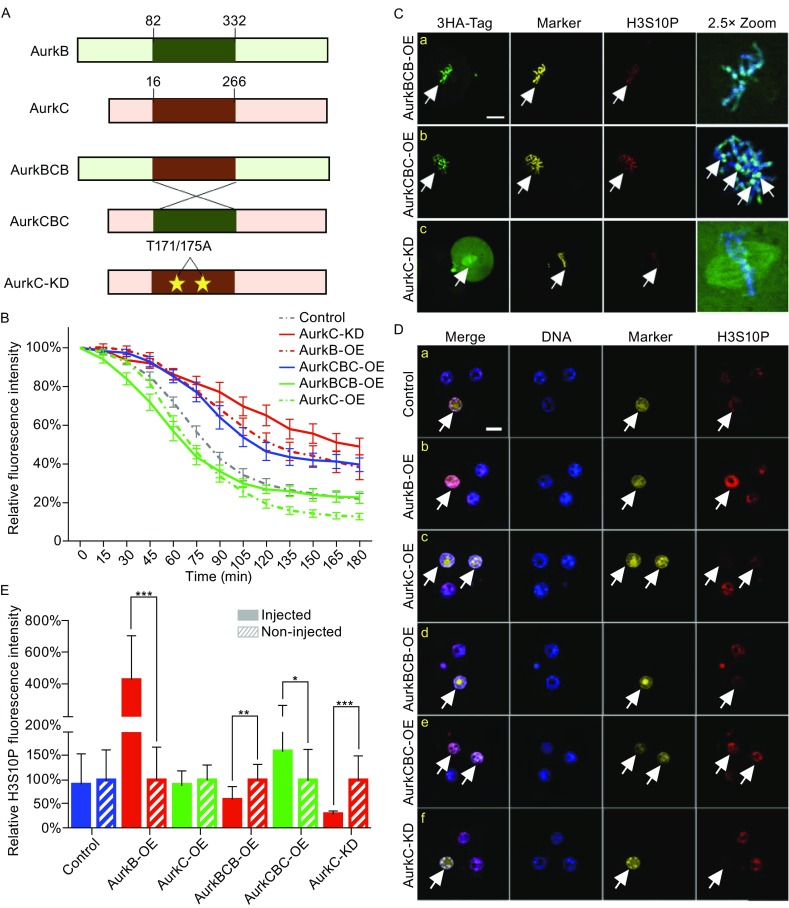
**The kinase domain of AurkB and AurkC determined their biological activity**. (A) Schematic view of the domain structure of AurkB, C, CBC, BCB, and kinase deficient AurkC-KD. (B) The Securin-mCherry fluorescence (Y-axis) degradation curve in AurkBCB-OE (*n* = 21), AurkCBC-OE (*n* = 22), and AurkC-KD (*n* = 26) groups, from NEBD to sister chromatids separation. Y-axis, fluorescence intensity; X-axis, time (min). The continuous line and whiskers indicate means and SEM. (C) 3HA-AurkBCB, CBC, and AurkC-KD localization during 2–4 cell stage division. DNA (blue), 3HA-tag (green), H2B-GFP (yellow), H3S10P (red). Scale bar, 20 μm. (D) H3S10P immunostaining of 4-cell stage embryos in the control (*n* = 26), AurkB-OE (*n* = 28), AurkC-OE (*n* = 24), AurkBCB-OE (*n* = 30), AurkCBC-OE (*n* = 22), and AurkC-KD (*n* = 24) groups. DNA (blue), H2B-GFP (marker, yellow), H3S10P (red). Scale bars, 20 μm. (E) Relative fluorescence intensity of H3S10P in injected and no injected cells. Control (*n* = 26), AurkB-OE (*n* = 28), AurkC-OE (*n* = 24), AurkBCB-OE (*n* = 30), AurkCBC-OE (*n* = 22), and AurkC-KD (*n* = 24) groups. The bar and whiskers indicate means and SD, *P < 0.05, **P < 0.01, ***P < 0.001

AurkB and AurkC shared similar domain structure. To better understand how AurkB and AurkC differentially regulate mitosis progression, we constructed kinase domain swapped AurkB/C chimeric proteins, namely AurkBCB and AurkCBC (Fig. 2A). We also generated a kinase dead AurkC (AurkC-KD) which carried T171A and T175A mutations in the cAMP-dependent protein kinase phosphorylation sites, the double mutants could abolish the activity of AurkC (Chen and Tang, 2002). The mRNAs of mutant Aurora kinases were mixed with Securin-mCherry and H2B-GFP mRNAs and microinjected into one blastomere of 2-cell stage embryos, followed by time-lapse imaging during 2 to 4-cell division as described in Fig. 1E. Quantification of Securin-mCherry degradation rate revealed that AurkCBC, which had the kinase domain from AurkB, showed delayed Securin-mCherry degradation similar with AurkB-OE. Cells took about 120 min (*n* = 21) to reach 50% of the Securin-mCherry fluorescence (Fig. 2B). While AurkBCB, which contained the AurkC kinase domain, showed accelerated degradation of Securin-mCherry. Cells expressing AurkBCB took about 75 min (*n* = 22) to reach 50% Securin level, which was similar to the degradation curve of AurkC-OE cells (Fig. 2B). Interestingly, the slope of the degradation curve in AurkCBC-OE cells was slightly steeper than that from AurkB-OE cells (Fig. 2B), suggesting that the AurkC domains flanking the kinase domain may also play some roles in dampening the checkpoint activity. In AurkBCB-OE cells, although the degradation of Securin-mCherry was initially faster than that in AurkC-OE cells, it did not reach as low level as in AurkC-OE cells (Fig. 2B), suggesting that the N- and C-terminus non-kinase domain of AurkB can elevate the AurkC kinase activity. The overexpression of AurkC-KD strongly inhibited Securin-mCherry degradation which reduced only 40% after 180 min (Fig. 2B). Consequently, nearly 90% (*n* = 26) of the cells that overexpressed AurkC-KD failed to complete cytokinesis (Movie S2). We examined the cellular localization of mutant Aurora kinases. Interestingly, AurkBCB showed similar location as AurkC during metaphase, it distributed along the chromosome (Fig. 2C), while AurkCBC appeared to concentrate more on the kinetochore/centromere region (Fig. 2C-b, arrow heads). In contrast, significant amount of AurkC-KD diffusely distributed in the cytoplasm, some of it associated with spindle microtubules and chromosomes (Fig. 2C-c). Above results suggest that the kinase domain was key to the unique function of AurkB and AurkC, namely, the specific chromosome location and differential regulation of SAC activity. As AurkC-KD failed to localize properly, it may sequester other key chromosome passenger proteins that required for the degradation of securin-mCherry thus lead to failure in sister chromatids separation.

In preimplantation embryos, low level of H3S10P was present during interphase. As H3S10 is a well-known substrate of AurkB phosphorylation during mitosis in somatic cells (Crosio et al., 2002). We also examined the effect of AurkB and AurkC towards interphase H3S10P. Immunostaining revealed that the overexpression of AurkB significantly up-regulated H3S10P level during interphase (Fig. 2D-b). Quantification showed more than 4-fold increase of H3S10P fluorescence intensity in AurkB-OE cells (*n* = 28), whereas AurkC-OE (*n* = 24) did not significantly affect H3S10P level at this stage (Fig. 2D-c and 2E). In contrary, AurkC-KD significantly reduced interphase H3S10P (Fig. 2D-f). The H3S10P intensity in AurkC-KD cells were reduced to about 70% of that in control cells (*n* = 24) (Fig. 2E). The level of interphase H3S10P seemed to positively correlate with the presence of AurkB kinase domain, as the chimeric AurkBCB reduced H3S10P level by about 40% (*n* = 30), while AurkCBC increased H3S10P level by 60% (*n* = 22) (Fig. 2D-a, 2D-b, and 2E). To discover whether AurkB/C may affect other important histone modifications in preimplantation embryos indirectly through differentially regulation of H3S10P level, we performed immunostaining of H3K9me3 and H3K4me3, which are histone marks of repressed and active chromatin respectively. They did not appear to be significantly affected by AurkB or AurkC (Figs. S2 and S3). Although H3K9me3 staining (Fig. S2A and S2B) showed a negative correlation with H3S10P, a recent paper reported that the affinity of H3K9me3 antibody can be affected by the adjacent H3S10 phosphorylation (Rothbart et al., 2015). Hirota et al. reported that H3S10 phosphorylation by AurkB could lead to HP1 dissociation from the heterochromatin (Hirota et al., 2005). However, HP1 expression pattern was similar in AurkB or AurkC overexpression cells (Fig. S2A).

### Aurora kinase B and C level affected Oct4 nuclear kinetics in cleavage stage embryos

**Figure 3:**
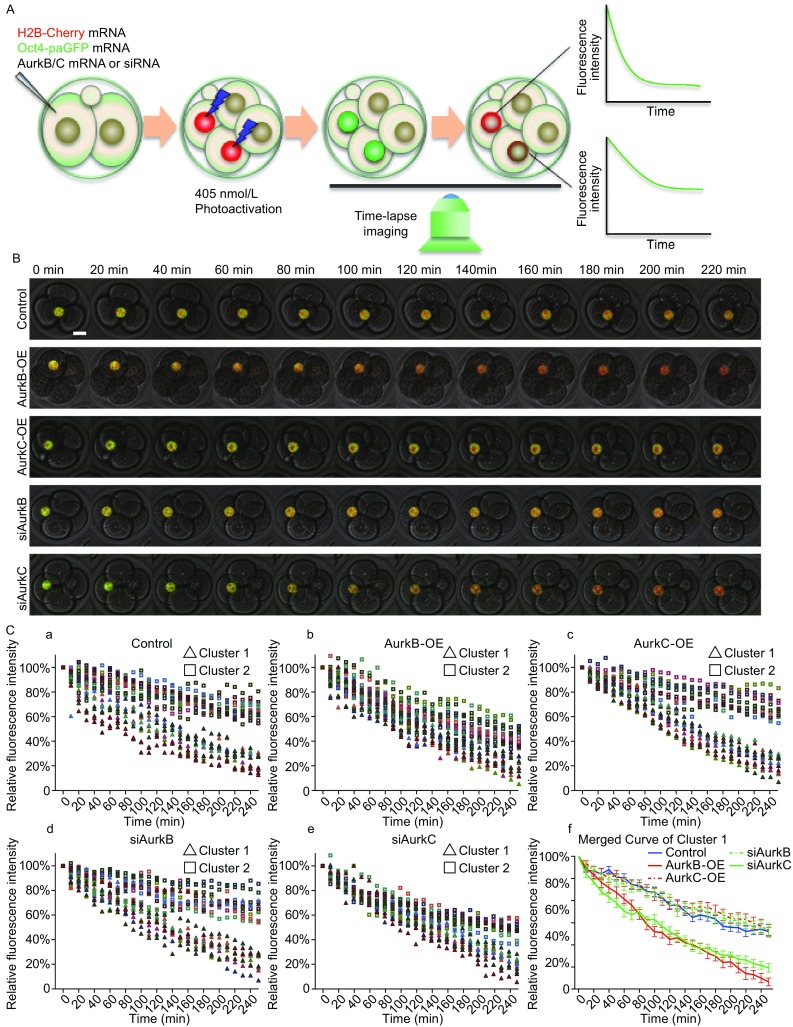
**Aurora kinase B and C level affected Oct4 kinetics in the nucleus**. (A) Schematic view of Oct4-paGFP fluorescence decay after photoactivation (FDAP) assay. (B) Selected frames from time lapse imaging movies of Oct4-paGFP degradation in control, AurkB-OE, AurkC-OE, siAurkB, and siAurkC groups embryos. H2B-mCherry (red), Oct4-paGFP (green), time interval, 10 min. Scale bars, 20 μm. (C) The Oct4-paGFP relative fluorescence (Y-axis) degradation analysis in control (*n* = 23), AurkB-OE (*n* = 26), AurkC-OE (*n* = 22), siAurkB (*n* = 20), and siAurkC (*n* = 23) groups, time interval 10 min, every symbol line represents an embryo in the group and the continuous line and whiskers in merged curve indicate means and SEM

We next tested whether the level of AurkB/C may affect the biological activity of key pluripotency factors. To this end, we constructed a photoactivable GFP fused to the C-terminus of Oct4 (Oct4-paGFP) similar to the one described by Plachta et al. (2011). We injected one blastomere of the late 2-cell stage embryo with a mRNA mixture of Oct4-paGFP, H2B-mCherry, and AurkB or AurkC. Embryos were cultured to mid-4-cell stage in the incubator, the two daughter cells of the injected cell can be recognized by the H2B-mCherry expression. We then performed the fluorescence decay after photoactivation assay (FDAP) using a confocal microscope (Fig. 3A). Upon targeted photoactivation of Oct4-paGFP by a 40% intensity of 405 nm laser for 3 s, the nucleus in the focus plan, which labelled with H2B-mCherry, showed bright green fluorescence, indicating successful conversion of the photoactivatable protein (Fig. S4A and S4B). Due to different focus plan, only the nucleus in focus appeared with fluorescence (Fig. S4C). Z-plans (2 μm/section, 31 sections in total) were acquired every 10 min for 4 h. The fluorescence intensity of Oct4-paGFP was quantified over time (Fig. 3B, 3C, and Movie S3A–E). The delay rate of Oct4-paGFP appeared to fall into two clusters: cluster 1, slower decay, and cluster 2, faster decay, which were likely to represent higher and lower affinity of Oct4 binding to DNA (Fig. 3C-a, control). This was similar to the previous report by Plachta et al. (2011). Interestingly, upon alteration of AurkB and C levels, cells from injected 4-cell embryos still had cluster 2 Oct4-paGFP decay. However, in AurkB-OE and siAurkC groups, fewer cells had cluster 1 decay or the decay rate of cluster 1 shifted towards cluster 2 (Fig. 3C-b and 3C-e). This suggests that increasing AurkB level or decreasing AurkC level may affect the kinetics of Oct4-DNA binding.

### Cells with higher AurkC level tend to enter the ICM lineage

**Figure 4:**
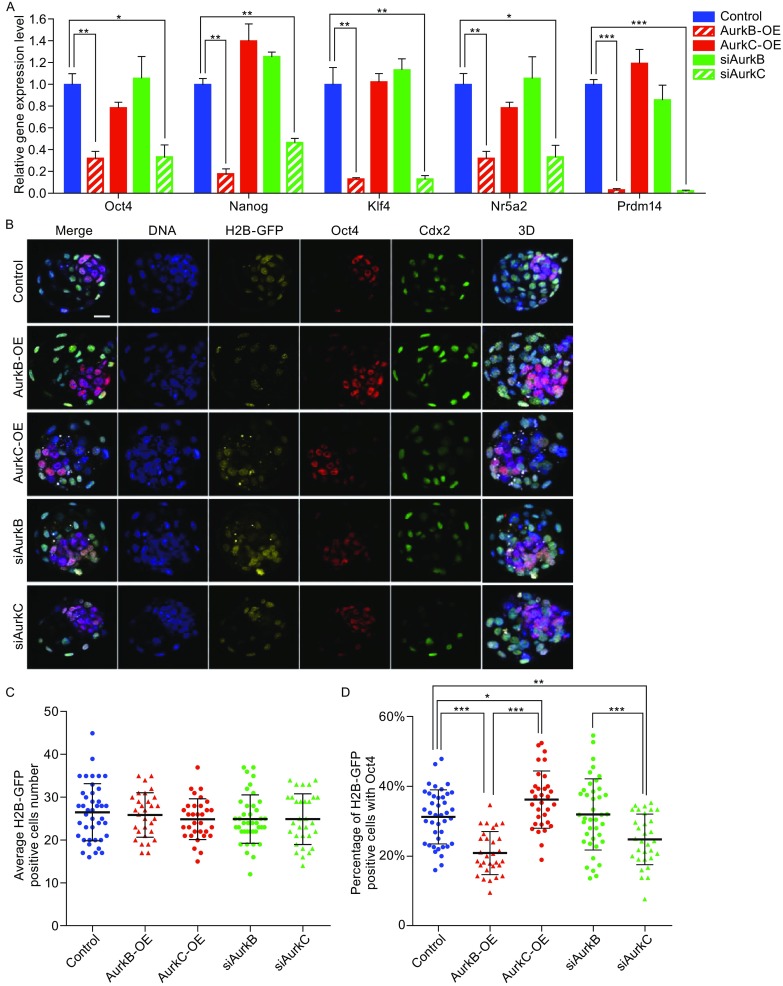
**Aurora kinase B and C affected pluripotency genes expression and cell fate during early morula stage**. (A) Relative genes expression analysis (control, AurkB-OE, AurkC-OE, siAurkB, siAurkC) of early morula stage (8-cell stage) embryos. Each sample were normalized by control, the bar and whiskers indicate means and SEM, *P < 0.05, **P < 0.01, ***P < 0.001. (B) Immunostaining of Oct4 and Cdx2 in late blastocyst stage of the control (*n* = 41), AurkB-OE (*n* = 30), AurkC-OE (*n* = 32), siAurkB (*n* = 40), and siAurkC (*n* = 31) groups. DNA (blue), H2B-GFP (marker, yellow), Oct4 (red), Cdx2 (green), 3D projections (max. proj.) of merged images are shown in the right panels. Scale bars, 20 μm. (C) Average cell number of H2B-GFP positive cell in the control (*n* = 41), AurkB-OE (*n* = 30), AurkC-OE (*n* = 32), siAurkB (*n* = 40), and siAurkC (*n* = 31) groups, each point represents data from one embryo, the bar and whiskers indicate means and SD, H2B-GFP positive cells are the daughter cells of the injected blastomere. (D) Percentage of H2B-GFP positive cells also expressed Oct4 in control (*n* = 41), AurkB-OE (*n* = 30), AurkC-OE (*n* = 32), siAurkB (*n* = 40), and siAurkC (*n* = 31) groups, each dot represents data from one embryo, the bar and whiskers indicate means and SD, *P < 0.05, ***P < 0.001

To investigate whether different levels of AurkB and AurkC may influence cell fate, we dissociated early 8-cell stage embryos into single cells and compared the pluripotency genes expression in the daughter cells divided from injected cells. In AurkB-OE and siAurkC embryos which had delayed cell cycle, the levels of pluripotency genes *Oct4*, *Nanog*, *Klf4*, *Prdm14*, and *Nr5a2* were significantly reduced (Fig. 4A). While in AurkC-OE and siAurkB cells, which had accelerated mitosis, the expression levels of above genes were similar with the control group (Fig. 4A).

To find out whether changing AurkB/C level may affect cell fate at blastocyst stage, we injected AurkB or AurkC mRNA/siRNA (with H2B-GFP as a marker) into one cell of 2-cell stage embryos and cultured them to blastocyst stage (E4.0). They were fixed and immunostained for Oct4 and Cdx2 proteins, followed by confocal microscopy analysis (Fig. 4B). We found that the total number of H2B-GFP positive cells was not significantly different in AurkB/C-OE or siAurkB/C groups, the average positive cell number was 26.54 ± 1.047 in control group (*n* = 41), 25.90 ± 0.9521 in AurkB-OE group (*n* = 30), 24.91 ± 0.8429 in AurkC-OE group (*n* = 32), 24.95 ± 0.8951 in siAurkB group (*n* = 40), and 24.94 ± 1.067 in siAurkC group (*n* = 31) (Fig. 4C). Interestingly, in AurkB-OE embryos, the percentage of Oct4 positive cells among H2B-GFP cells was clearly reduced (20.86% ± 1.13, *n* = 30) compared to that in control embryos (31.23% ± 1.21, *n* = 41) (Fig. 4D). While higher percentage of daughter cells from AurkC-OE cells had Oct4 expression (36.18% ± 1.45, *n* = 32). AurkB knock-down did not affect the ratio of Oct4 positive cells (31.92% ± 1.61, *n* = 40), but AurkC knock-down lead to fewer Oct4 positive cells amongst H2B-GFP cells (24.76% ± 1.30, *n* = 31), which was similar to AurkB-OE.

**Figure 5:**
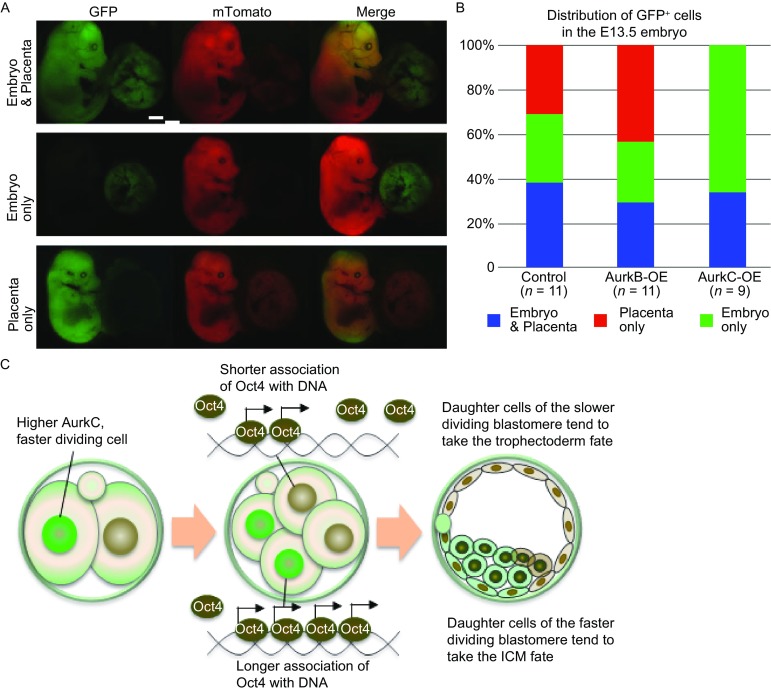
**Long term effect of AurkB and AurkC overexpression during preimplantation embryo development**. (A) Images of GFP positive cells in E13.5 Rosa embryos and placenta. The distribution was listed at the side of images. Scale bars, 2 mm. (B) Bar graph quantification of the percentage of embryo or placenta or both. Control (*n* = 11), AurkB-OE (*n* = 11), AurkC-OE (*n* = 9) groups. (C) Model of how cell division may affect cell fate decision in preimplantation mouse embryos

Next, we used ROSA26Sor^tm4(ACTB-tdTomato, -EGFP) Luo^ (ROSA) transgenic mice to trace the lineage of AurkB/C-OE cells. H2B-GFP, AurkB or AurkC mRNAs were co-injected with CRE mRNA into one cell of 4-cell stage embryos from ROSA female mice crossed with F1 male mice. Upon CRE protein expression, it will excise the membrane-bound Tomato (mTomato) cassette to allow the expression of the downstream membrane-bound GFP (mGFP), thus the progeny of the cell where CRE was expressed will display mGFP fluorescence, while the rest of the embryo will have mTomato red fluorescence. After injection, embryos were transferred into the oviduct of pseudopregnant ICR female mice. At E13.5, embryos were dissected for observation. Three types of GFP cell distribution can be observed: located only to the embryo body, or only to the placenta, or to both embryo body and placenta (Fig. 5A). Progenies derived from cells with transient AurkB overexpression were more likely to contribute to the placenta (5/11) and less likely to the embryo body (2/11), while cell descendent from transient AurkC overexpression blastomere preferred to contribute to embryo body (6/9) (Fig. 5B). These results suggested that blastomeres with higher AurkC level indeed were more likely to take the pluripotent cell fate and contribute to the embryo development.

Taken together, our results suggested that the levels of AurkB and C in cleavage stage embryos may influence cell division speed as well as histone H3S10 phosphorylation level, and subsequently affect cell fate choice during development.

## Discussion

In this study, by modulation of AurkB or AurkC levels in part of mouse preimplantation embryo, combined with live cell imaging and lineage tracing experiments, we showed that changing key mitotic kinase activities can significantly impact the cell division dynamics and cell fate decision.

Previous research demonstrated that in both mouse and human cleavage stage embryos, AurkC was present at much higher level than AurkB, a ubiquitously expressed mitotic kinase (Avo Santos et al., 2011; Fernandez-Miranda et al., 2011; Schindler et al., 2012; Sharif et al., 2010). Despite their similarities in the protein structure and cellular localization at chromosome passenger complex, AurkC displayed unique characteristics different from AurkB. Lin et al. showed that in cancer cells, overexpression of AurkC could displace AurkB from centromere and impaired SAC function (Lin et al., 2014). Our previous results also showed that high level AurkC in mouse oocytes accelerated securin degradation, caused chromosome mis-alignment and cytokinesis failure (Sharif et al., 2010). The mitosis in cleavage mammalian embryos are particularly error prone, consequently, a high proportion of chromosomal mosaicism was observed (Avo Santos et al., 2011; Vanneste et al., 2009). Curiously, AurkC but not AurkB was higher expressed during this time window, and AurkC expression level was higher in the daughter cells from the earlier dividing blastomere of the 2-cell embryo. Using a live-imaging based securin degradation assay, we showed that increasing AurkC level advanced securin destruction, while knocking down AurkC had the opposite effect. In contrast, more AurkB protein correlated with slower securin degradation and vice versa. The degradation rate of securin can be considered as a readout of SAC and APC/C activity (Hagting et al., 2002). Thus, our results imply that the relatively high level of AurkC may create a more “relaxed” SAC state that adapts to the cleavage stage embryos. While AurkB represents a “stricter” SAC which is normally present in somatic cells. It has been proposed that after fertilization, it may take several cell cycles to transform all the components of cell division from meiosis to mitosis (Clift and Schuh, 2013). Moreover, upon fertilization, there’s dramatic epigenetic reprogramming to create an open chromatin state that permits the robust zygotic genome activation during the first few mitotic cell cycles (Clift and Schuh, 2013). The special chromatin modifications may be recognized as abnormal by cell cycle checkpoints and activate stress response pathway. Therefore, we speculate it may be necessary for cleavage stage embryos to evolve a special cell division checkpoint mechanism, AurkC may be an important member of such mechanism.

It has been shown that the kinase activity is essential for the function of AurkB (Krenn and Musacchio, 2015). To have a deeper understanding of the molecular basis that leads to distinct functions of AurkB and C, we generated series of mutant Aurora kinases: AurkBCB, AurkCBC, and the kinase dead AurkC-KD. We found that the degradation rate of securin was largely determined by the AurkB/C kinase domain (Fig. 2B). H3S10 is a well-known substrate of AurkB (Crosio et al., 2002). In this study, we examined both metaphase and interphase H3S10P to evaluate the kinase activity of AurkB/C series of constructs. Strong H3S10P during metaphase can be observed in cells injected with either wild type or mutant Aurora kinases including AurkC-KD (Fig. 2C). Interestingly, the interphase H3S10P was significantly affected by the kinase domain overexpressed. Specifically, constructs with AurkB kinase domain (AurkB and AurkCBC) significantly increased H3S10P, whereas AurkBCB which has the AurkC kinase domain flanked by the AurkB N- and C-terminus reduced interphase H3S10P. These results strongly suggest that kinase domain of AurkC may have weaker kinase activity than that of AurkB (Fig. 2D and 2E). Consistent with this hypothesis, when AurkC-KD was overexpressed, interphase H3S10P was markedly diminished (Fig. 2D and 2E). The kinase dead mutations also lead to significantly slower Securin degradation (Fig. 2B), this was likely due to severely mis-localized AurkC-KD protein during mitosis (Fig. 2C) which inhibited the function of APC/C. Similar phenotype was observed in mouse oocytes ectopically expressed kinase dead AurkC (Yang et al., 2010).

The ability of AurkB/C to regulate interphase H3S10P leads us to ask whether they have different impact on transcription factor activity, gene expression, and cell fate decisions. Indeed, both cell division timing and histone modifications have been shown to affect cell fate decision in preimplantation embryos (Zernicka-Goetz, 2006). Recently, AurkB was reported to phosphorylate Oct4 on S229 and reduced its binding ability to target genes in mouse embryonic stem cells (mESCs) (Shin et al., 2016). In our experiments, we utilized a Oct4-paGFP construct and FDAP assay (Plachta et al., 2011) to investigate how AurkB and AurkC variation may affect Oct4 kinetics. We found that raising AurkB level caused faster Oct4-paGFP decay in the nucleus which is likely to reflect reduced affinity of Oct4 binding to DNA (Fig. 3C). Gene expression analysis also confirmed that the expression of several Oct4 target genes were decreased when AurkB level was high (Fig. 4A). Although we didn’t observe that AurkC altered the kinetics of Oct4-paGFP decay in 4-cell embryos, knocking-down AurkC by siRNA significantly reduced the expression of pluripotency marker genes (Fig. 4A). Moreover, more daughter cells from the AurkC-OE cell expressed Oct4 and preferably occupied ICM position (Fig. 4B and 4D). In contrast, significant fewer daughter cells from AurkB-OE or siAurkC cell expressed Oct4 and entered ICM lineage (Fig. 4B and 4D). Lineage tracing experiments also confirmed that progeny cells derived from the AurkC-OE blastomere at 4-cell stage tend to contribute to the embryo proper (Fig. 5A and 5B).

In summary, our results demonstrated that it is possible to control cell fate by transiently modulating the level of AurkB and C in preimplantation embryos. We proposed a model as depicted in Fig. 5C, during preimplantation development, key cell division kinases such as AurkB and C may differentially regulate mitosis timing and the activities of pluripotency transcription factors thus to influence cell position, gene expression and these effects cumulate to impact cell fate decisions. Our study also leads to more questions, what are the factors that affect the activity of cell division regulators in preimplantation embryos, is it just the stochastic fluctuation or inherited from oocytes? Will different interphase H3S10P level lead to additional change in histone modification? As chromosome abnormality caused by erroneous mitosis can lead to severe consequences, mRNA mediated transient modulation of cell division checkpoints or epigenetic state may represent a possible route to improve the quality of *in vitro* fertilized mammalian embryos without alteration of the embryo genome.

## Materials and methods

### Embryo collection, culture, and microinjection

All animal experiments were conducted in accordance with the Guide for the Care and Use of Animals for Research Purposes. The protocol for mouse embryo isolation was approved by Institutional Animal Care and Use Committee and Internal Review Board of Tsinghua University.

Oocytes and embryos were collected from wild type F1 (C57BL/6xDBA) females (Charles River) as previously described (Na and Zernicka-Goetz, 2006). ROSA26Sor^tm4(ACTB-tdTomato, -EGFP) Luo^ transgenic mice (JAX stock number 007676) were obtained from Jackson laboratory and maintained as homozygotes. Zygotes for mRNA injections were collected from female mice 25–26 h post-hCG. 2-, 4-, and 8-cell embryos were collected from female mice 46, 56 or 64 h post-hCG, respectively. Morula and blastocysts were collected at 2.5 dpc or 4 dpc, respectively. Microinjection of mRNA and siRNA into mouse preimplantation embryos were performed on a Leica DMI3000B microscope equipped with a Leica micromanipulator as previously described (Na and Zernicka-Goetz, 2006) at desired stages.

### Plasmid construction, mRNA synthesis, and siRNA preparation

HA tagged (N-terminus) AurkB and AurkC, Securin-mCherry, H2B-GFP or mCherry and Oct4-paGFP were engineered and subcloned into RN3P vector for *in vitro* transcription of mRNA. Capped mRNAs were generated using a T3 mMESSAGE mMACHINE Kit according to the manufacture’s instructions (AM1348, Ambion/Thermo Fisher Scientific). SiRNA targeting Aurora B and C were designed and purchased from siRNA Design Service (Sigma). SiRNA with scrambled sequence was used as the control.

### Embryo fixation and immunostaining

For immunostaining, mouse preimplantation embryos were first treated with Acidic Tyrode solution to remove the zona pellucida. Then the embryos were fixed with 1% PFA in PBS in 4°C overnight. After fixation, embryos were permeabilized with 0.25% Triton X-100 at room temperature for 20 min and blocked with 3% BSA in PBS at 4°C overnight. Primary antibodies incubation was carried out in 4°C overnight. The primary antibodies include: monoclonal mouse anti-HA (ab130275, Abcam), monoclonal rat anti-αTubulin (sc-53029, Santa Cruz), monoclonal rabbit anti-H3S10P (#9701S, CST), polyclonal rabbit anti-Oct4 (ab19857, Abcam), monoclonal mouse anti-Cdx2 (CDX2-88, Biogenex). Then the samples were incubated with DyLight 488/549/633 conjugated Goat anti mouse or rabbit IgG (H + L) antibodies (#35502, #35557, #35512, Thermo) at 4°C overnight, and nucleus were stained with DAPI. After staining, embryos were mounted on coverslips in Vectashield mounting medium (H-1000, Vectashield) and imaged on Nikon A1 confocal microscope.

### Q-PCR

Embryos were first lysed in 0.2% Triton X-100 solution. Then reverse transcription and amplification of cDNA were performed using the Smart-seq2 protocol (Picelli et al., 2014). Q-PCR reactions and analysis were performed with GoTaq Q-PCR Master Mix (A6002, Promega) on a Bio-rad CFX96 Q-PCR Amplifier. The primer sequences and annealing temperature are listed in Table S1.

### Time-lapse imaging experiments

Securin-mCherry degradation experiment was performed on a Leica AF6000 inverted microscope with a 20× objective (NA = 0.7). The microscope was equipped with a PECON environmental control chamber, fully motorized stage (VETXA) with plate heating insert (PECON). Embryos were cultured to 37°C and 5% CO_2_ atmosphere. Oct4-paGFP FDAP assay were performed on a Nikon A1 confocal microscope equipped with TOKAI HIT atmospheric chamber using a method modified from Plachta et al. (2011). In brief, at 4-cell stage, 2 daughter cells of the injected blastomere from the 2-cell embryo were identified based on the expression of H2B-mCherry. Their nuclear regions were selectively photoactivated using the 405 nm laser within 5 min. Then z-series scan (2 μm per section) was performed every 10 min for up to 4 h across each nuclear region.

### Embryo transfer into foster female mice and postimplantation embryo dissection

Foster female mice (F1) were crossed with ligated male mice 0.5 day before embryo transfer operation. Successful mating was determined by the presence of vaginal plug the next morning. At 1.5 dpc, plugged pseudo-pregnant female mice were anesthetized, CRE and AurkB/C mRNA injected ROSA 4-cell stage embryos were transferred into the oviduct using glass transfer pipette and the mice were nursed until vivification. The operated mice were sacrificed after 12 days, post-implantation embryos with placenta (13.5 dpc equivalent) were isolated from the uterus and stripped off the extraembryonic membranes. The embryo proper with the placenta were imaged on a Leica M165 FC integrated microscope.

## Electronic supplementary material

Below is the link to the electronic supplementary material.

Supplementary material 1 (AVI 412 kb)

Supplementary material 2 (AVI 412 kb)

Supplementary material 3 (AVI 471 kb)

Supplementary material 4 (AVI 471 kb)

Supplementary material 5 (AVI 412 kb)

Supplementary material 6 (AVI 1623 kb)

Supplementary material 7 (AVI 1705 kb)

Supplementary material 8 (AVI 1705 kb)

Supplementary material 9 (AVI 1705 kb)

Supplementary material 10 (AVI 1705 kb)

Supplementary material 11 (AVI 1705 kb)

Supplementary material 12 (PDF 461 kb)

Supplementary material 13 (PDF 66 kb)

Supplementary material 14 (PDF 69 kb)

Supplementary material 15 (PDF 66 kb)

## References

[CR1] Santos MA, van de Werken C, de Vries M, Jahr H, Vromans MJ, Laven JS, Fauser BC, Kops GJ, Lens SM, Baart EB (2011). A role for Aurora C in the chromosomal passenger complex during human preimplantation embryo development. Hum Reprod.

[CR2] Balboula AZ, Schindler K (2014). Selective disruption of aurora C kinase reveals distinct functions from aurora B kinase during meiosis in mouse oocytes. PLoS Genet.

[CR3] Bolton H, Graham SJ, Van der Aa N, Kumar P, Theunis K, Fernandez Gallardo E, Voet T, Zernicka-Goetz M (2016). Mouse model of chromosome mosaicism reveals lineage-specific depletion of aneuploid cells and normal developmental potential. Nat Commun.

[CR4] Carmena M, Earnshaw WC (2003). The cellular geography of aurora kinases. Nat Rev Mol Cell Biol.

[CR5] Chazaud C, Yamanaka Y (2016). Lineage specification in the mouse preimplantation embryo. Development.

[CR6] Chen SH, Tang TK (2002). Mutational analysis of the phosphorylation sites of the Aie1 (Aurora-C) kinase in vitro. DNA Cell Biol.

[CR7] Clift D, Schuh M (2013). Restarting life: fertilization and the transition from meiosis to mitosis. Nat Rev Mol Cell Biol.

[CR8] Crosio C, Fimia GM, Loury R, Kimura M, Okano Y, Zhou H, Sen S, Allis CD, Sassone-Corsi P (2002). Mitotic phosphorylation of histone H3: spatio-temporal regulation by mammalian aurora kinases. Molecular and Cellular Biology.

[CR9] Dieterich K, Soto Rifo R, Faure AK, Hennebicq S, Ben Amar B, Zahi M, Perrin J, Martinez D, Sele B, Jouk PS, Ohlmann T, Rousseaux S, Lunardi J, Ray PF (2007). Homozygous mutation of AURKC yields large-headed polyploid spermatozoa and causes male infertility. Nat Genet.

[CR10] Fernandez-Miranda G, Trakala M, Martin J, Escobar B, Gonzalez A, Ghyselinck NB, Ortega S, Canamero M, Perez de Castro I, Malumbres M (2011). Genetic disruption of aurora B uncovers an essential role for aurora C during early mammalian development. Development.

[CR11] Hagting A, Den Elzen N, Vodermaier HC, Waizenegger IC, Peters JM, Pines J (2002). Human securin proteolysis is controlled by the spindle checkpoint and reveals when the APC/C switches from activation by Cdc20 to Cdh1. J Cell Biol.

[CR12] Hirota T, Lipp JJ, Toh BH, Peters JM (2005). Histone H3 serine 10 phosphorylation by Aurora B causes HP1 dissociation from heterochromatin. Nature.

[CR13] Krenn V, Musacchio A (2015). The aurora B kinase in chromosome bi-orientation and spindle checkpoint signaling. Front Oncol.

[CR14] Lin BW, Wang YC, Chang-Liao PY, Lin YJ, Yang ST, Tsou JH, Chang KC, Liu YW, Tseng JT, Lee CT, Lee JC, Hung LY (2014). Overexpression of Aurora-C interferes with the spindle checkpoint by promoting the degradation of Aurora-B. Cell Death Dis.

[CR15] Musacchio A (2015). The molecular biology of spindle assembly checkpoint signaling dynamics. Curr Biol.

[CR16] Na J, Zernicka-Goetz M (2006). Asymmetric positioning and organization of the meiotic spindle of mouse oocytes requires CDC42 function. Curr Biol.

[CR17] Nasmyth K (2005). How do so few control so many?. Cell.

[CR18] Picelli S, Faridani OR, Bjorklund AK, Winberg G, Sagasser S, Sandberg R (2014). Full-length RNA-seq from single cells using Smart-seq2. Nat Protoc.

[CR19] Plachta N, Bollenbach T, Pease S, Fraser SE, Pantazis P (2011). Oct4 kinetics predict cell lineage patterning in the early mammalian embryo. Nat Cell Biol.

[CR20] Rothbart SB, Dickson BM, Raab JR, Grzybowski AT, Krajewski K, Guo AH, Shanle EK, Josefowicz SZ, Fuchs SM, Allis CD, Magnuson TR, Ruthenburg AJ, Strahl BD (2015). An interactive database for the assessment of histone antibody specificity. Mol Cell.

[CR21] Ruchaud S, Carmena M, Earnshaw WC (2007). Chromosomal passengers: conducting cell division. Nat Rev Mol Cell Biol.

[CR22] Sasai K, Katayama H, Stenoien DL, Fujii S, Honda R, Kimura M, Okano Y, Tatsuka M, Suzuki F, Nigg EA, Earnshaw WC, Brinkley WR, Sen S (2004). Aurora-C kinase is a novel chromosomal passenger protein that can complement Aurora-B kinase function in mitotic cells. Cell Motil Cytoskeleton.

[CR23] Schindler K, Davydenko O, Fram B, Lampson MA, Schultz RM (2012). Maternally recruited Aurora C kinase is more stable than Aurora B to support mouse oocyte maturation and early development. Proc Natl Acad Sci USA.

[CR24] Schvartzman JM, Sotillo R, Benezra R (2010). Mitotic chromosomal instability and cancer: mouse modelling of the human disease. Nat Rev Cancer.

[CR25] Sharif B, Na J, Lykke-Hartmann K, McLaughlin SH, Laue E, Glover DM, Zernicka-Goetz M (2010). The chromosome passenger complex is required for fidelity of chromosome transmission and cytokinesis in meiosis of mouse oocytes. J Cell Sci.

[CR26] Shin J, Kim TW, Kim H, Kim HJ, Suh MY, Lee S, Lee HT, Kwak S, Lee SE, Lee JH, Jang H, Cho EJ, Youn HD (2016). Aurkb/PP1-mediated resetting of Oct4 during the cell cycle determines the identity of embryonic stem cells. Elife.

[CR27] Tabansky I, Lenarcic A, Draft RW, Loulier K, Keskin DB, Rosains J, Rivera-Feliciano J, Lichtman JW, Livet J, Stern JN, Sanes JR, Eggan K (2013). Developmental bias in cleavage-stage mouse blastomeres. Curr Biol.

[CR28] Vanneste E, Voet T, Le Caignec C, Ampe M, Konings P, Melotte C, Debrock S, Amyere M, Vikkula M, Schuit F, Fryns JP, Verbeke G, D’Hooghe T, Moreau Y, Vermeesch JR (2009). Chromosome instability is common in human cleavage-stage embryos. Nat Med.

[CR29] Wong CC, Loewke KE, Bossert NL, Behr B, De Jonge CJ, Baer TM, Reijo Pera RA (2010). Non-invasive imaging of human embryos before embryonic genome activation predicts development to the blastocyst stage. Nat Biotechnol.

[CR30] Xue Z, Huang K, Cai C, Cai L, Jiang CY, Feng Y, Liu Z, Zeng Q, Cheng L, Sun YE, Liu JY, Horvath S, Fan G (2013). Genetic programs in human and mouse early embryos revealed by single-cell RNA sequencing. Nature.

[CR31] Yang KT, Li SK, Chang CC, Tang CJ, Lin YN, Lee SC, Tang TK (2010). Aurora-C kinase deficiency causes cytokinesis failure in meiosis I and production of large polyploid oocytes in mice. Mol Biol Cell.

[CR32] Zernicka-Goetz M (2006). The first cell-fate decisions in the mouse embryo: destiny is a matter of both chance and choice. Curr Opin Genet Dev.

[CR33] Zernicka-Goetz M, Morris SA, Bruce AW (2009). Making a firm decision: multifaceted regulation of cell fate in the early mouse embryo. Nat Rev Genet.

