# Supplementary material for: Differential regulation of H3S10 phosphorylation, mitosis progression and cell fate by Aurora Kinase B and C in mouse preimplantation embryos
Source: Protein Cell. 2017 Apr 22;8(9):662–74. doi: 10.1007/s13238-017-0407-5 (PMC5563281; doi:10.1007/s13238-017-0407-5)
Supplement: Supplementary file 12 — Supplementary material 12 (PDF 461 kb) [file 13238_2017_407_MOESM12_ESM.pdf]

1 **Supplementary Figure Legends**

Figure S1

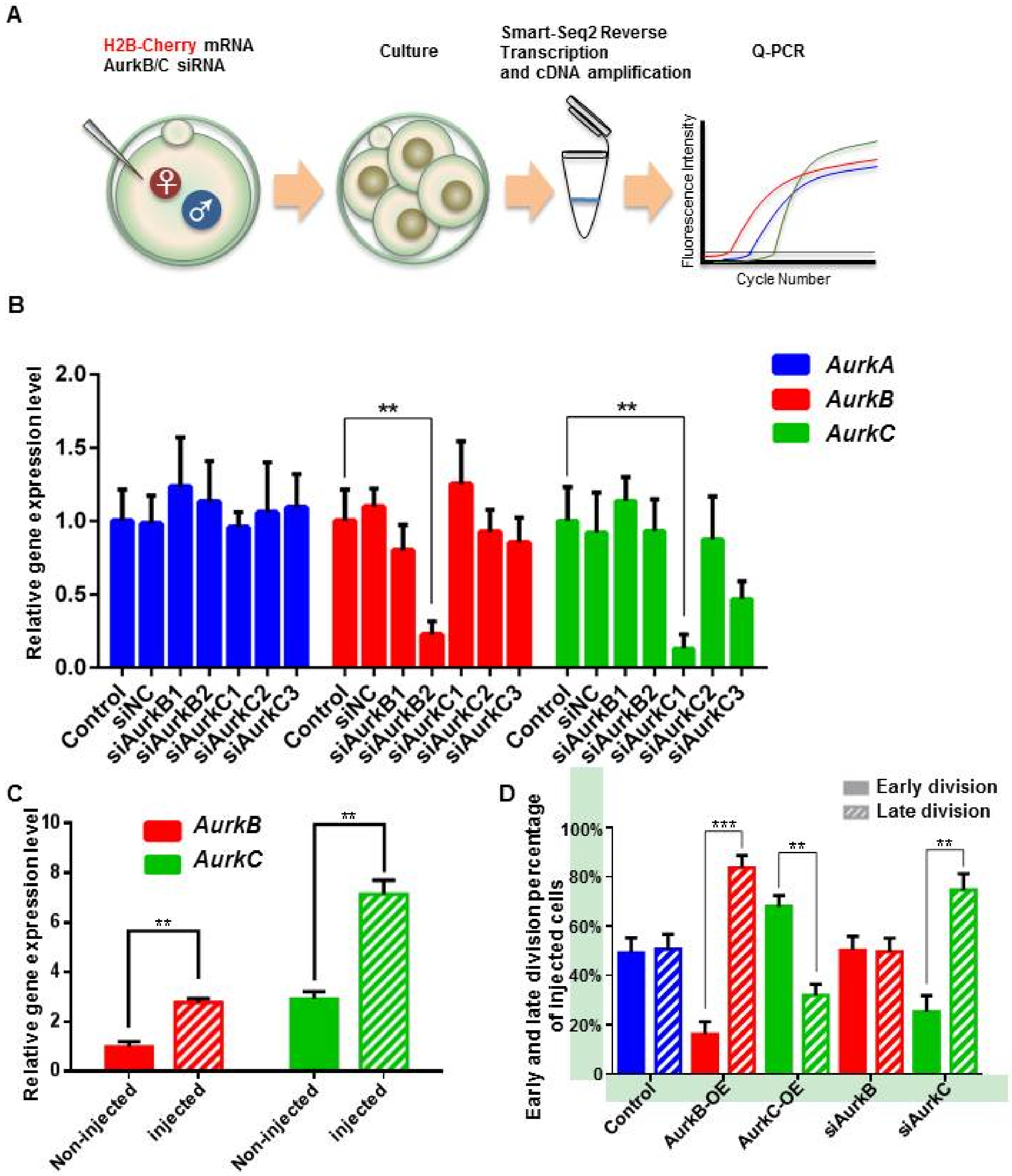

2

Figure S1. AurkB and AurkC siRNA knock-down efficiency confirmation in preimplantation embryos.

(A) Schematic view of AurkB and AurkC siRNA microinjection and Q-PCR confirmation of knocking-down efficiency in preimplantation mouse embryos.

(B) Relative genes expression analysis. SiRNAs (Control, siNC, siAurkB1, siAurkB2, siAurkC1, siAurkC2, siAurkC3) were injected at 1-cell sate, early morula stage (8-cell stage) embryos were collected, lysed and subjected to RT-QPCR analysis. The level of AurkA, B and C in the siControl injected embryos was considered “1”, the bar and whiskers indicate means and SEM, $^{**}p < 0.01$.

(C) Q-PCR analysis of AurkB and AurkC expression level before and after mRNA injection. mRNAs were injected at zygote stage, at early 4-cell stage, embryos were collected, lysed and subjected to RT-QPCR analysis. The level of AurkB in wild type embryos was considered “1”, the bar and whiskers indicate means and SEM, $^{**}p < 0.01$.

(D) Early and late division percentage of injected cells in the experiment of Figure 1E-H were counted, the bar and whiskers indicate means and SD, $^{**}p < 0.01$, $^{***}p < 0.001$.

Figure S2

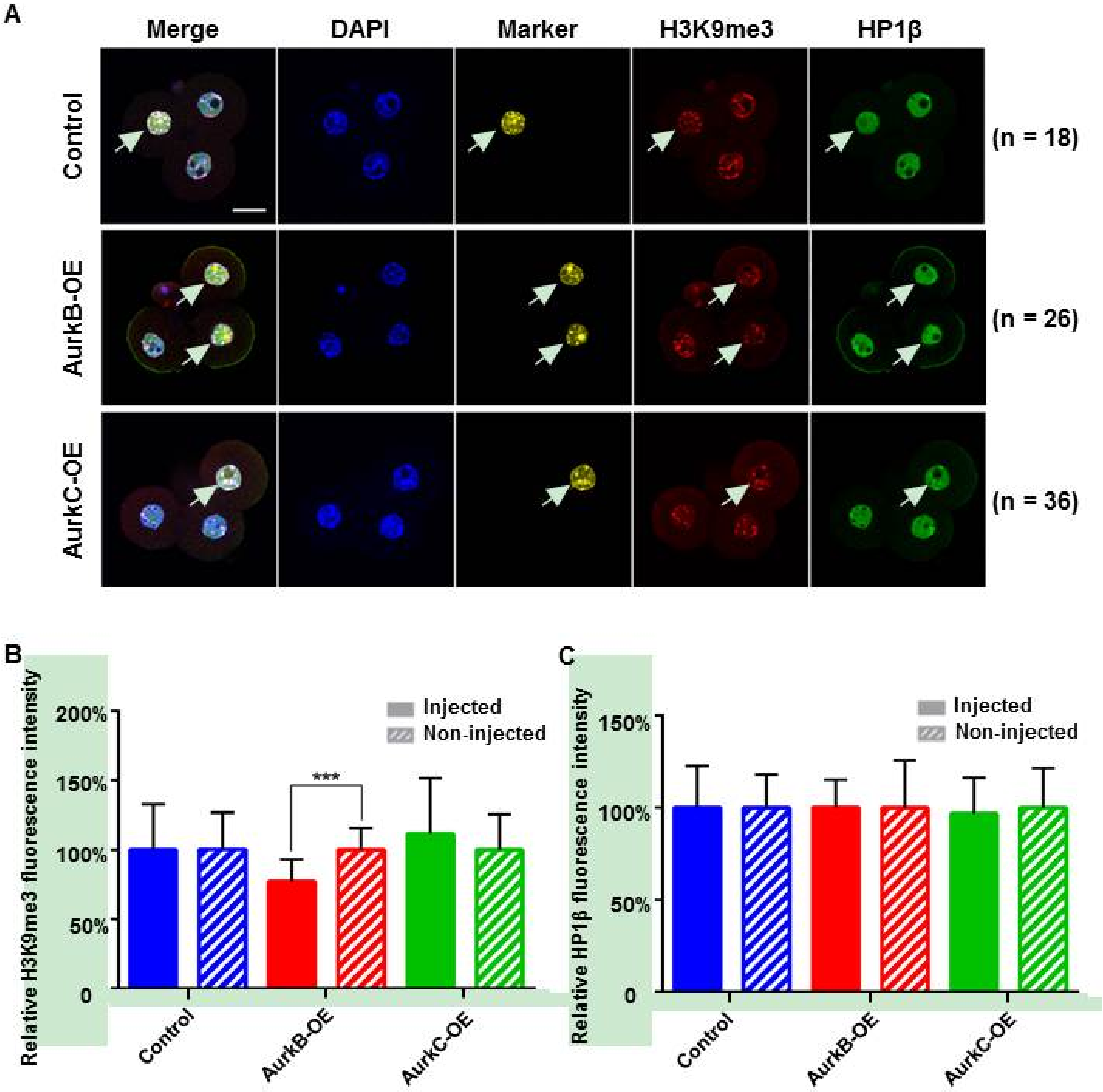
A
Merge
DAPI
Marker
H3K9me3
HP1β
Control
AurkB-OE
AurkC-OE
(n = 18)
(n = 26)
(n = 36)
B
Relative H3K9me3 fluorescence intensity
200%
150%
100%
50%
0
***
Injected
Non-injected
Control
AurkB-OE
AurkC-OE
C
Relative HP1β fluorescence intensity
150%
100%
50%
0
Injected
Non-injected
Control
AurkB-OE
AurkC-OE

18

Figure S2. Immunostaining of H3K9me3 and HP1β with AurkB and AurkC overexpression during 4-cell stage.

(A) H3K4me3 and H3S10P immunostaining of morula stage embryos in Control (n = 18), AurkB-OE (n = 26), AurkC-OE (n = 36) groups. DNA (blue), H2B-GFP (Marker, yellow), H3K9me3 (red), HP1β (green). Scale bars, 20 μm.

(B) Bar graph quantification of relative fluorescence intensity of H3K9me3 in injected and non-injected cells of Control (n = 18), AurkB-OE (n = 26), AurkC-OE (n = 36) groups. The bar and whiskers indicate means and SD, ***$p < 0.001$.

(C) Bar graph quantification of relative fluorescence intensity of HP1β in injected and non-injected cells of Control (n = 18), AurkB-OE (n = 26), AurkC-OE (n = 36) groups. The bar and whiskers indicate means and SD.

Figure S3

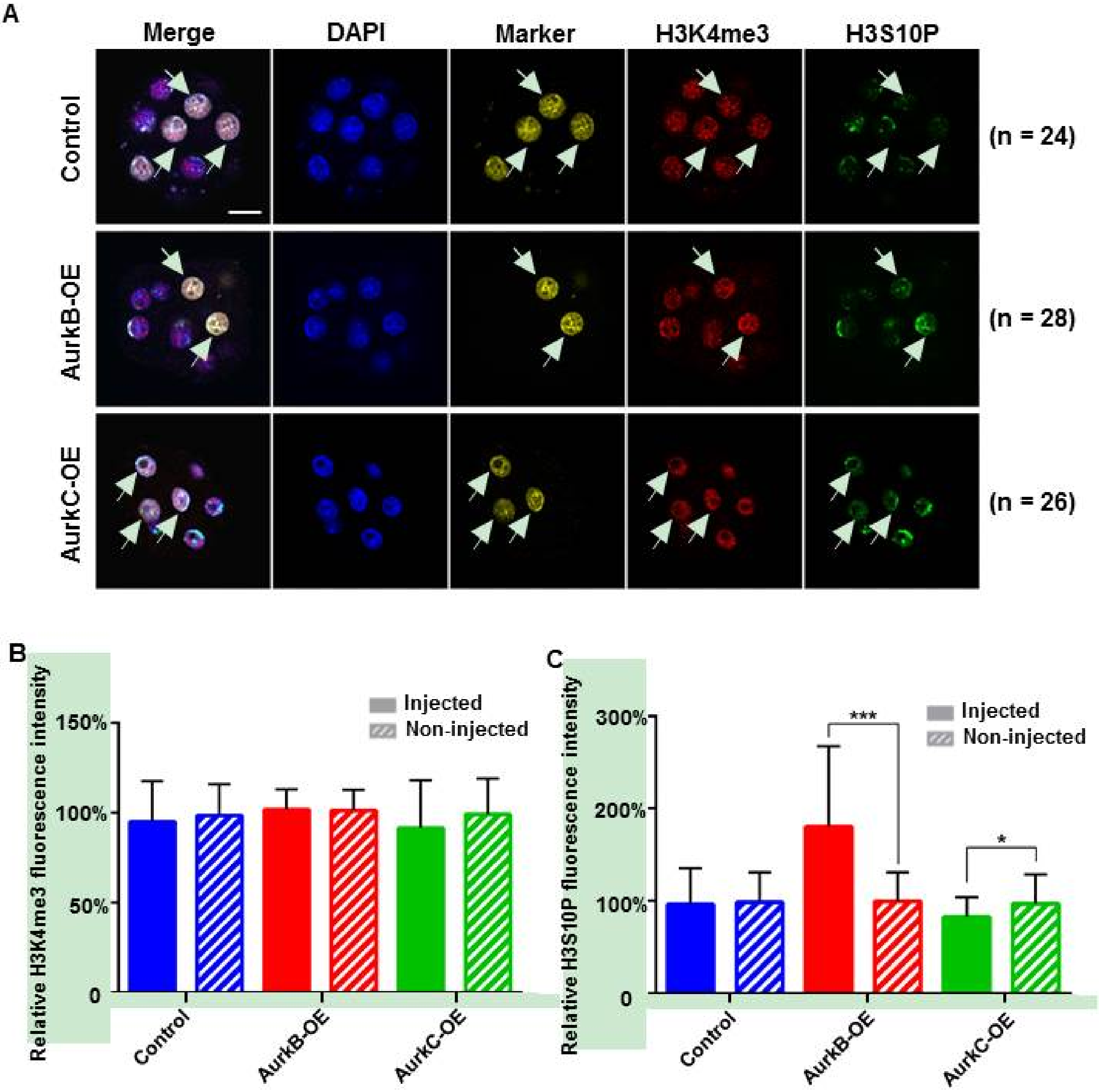
A
Merge
DAPI
Marker
H3K4me3
H3S10P
Control
AurkB-OE
AurkC-OE
(n = 24)
(n = 28)
(n = 26)
B
Relative H3K4me3 fluorescence intensity
150%
100%
50%
0
Injected
Non-injected
Control
AurkB-OE
AurkC-OE
C
Relative H3S10P fluorescence intensity
300%
200%
100%
0
***
*
Injected
Non-injected
Control
AurkB-OE
AurkC-OE

30

Figure S3. Immunostaining of H3K3me3 and H3S10P with AurkB and AurkC overexpression during morula stage.

(A) H3K4me3 and H3S10P immunostaining of morula stage embryos in Control (n = 24), AurkB-OE (n = 28), AurkC-OE (n = 26) groups. DNA (blue), H2B-GFP (Marker, yellow), H3K4me3 (red), H3S10P (green). Scale bars, 20 μm.

(B) Bar graph quantification of relative fluorescence intensity of H3K4me3 in injected and non-injected cells of Control (n=24), AurkB-OE (n = 28), AurkC-OE (n = 26) groups. The bar and whiskers indicate means and SD.

(C) Bar graph quantification of relative fluorescence intensity of H3S10P in injected and non-injected cells of Control (n=24), AurkB-OE (n = 28), AurkC-OE (n = 26) groups. The bar and whiskers indicate means and SD, *$p < 0.05$, ***$p < 0.001$.

Figure S4

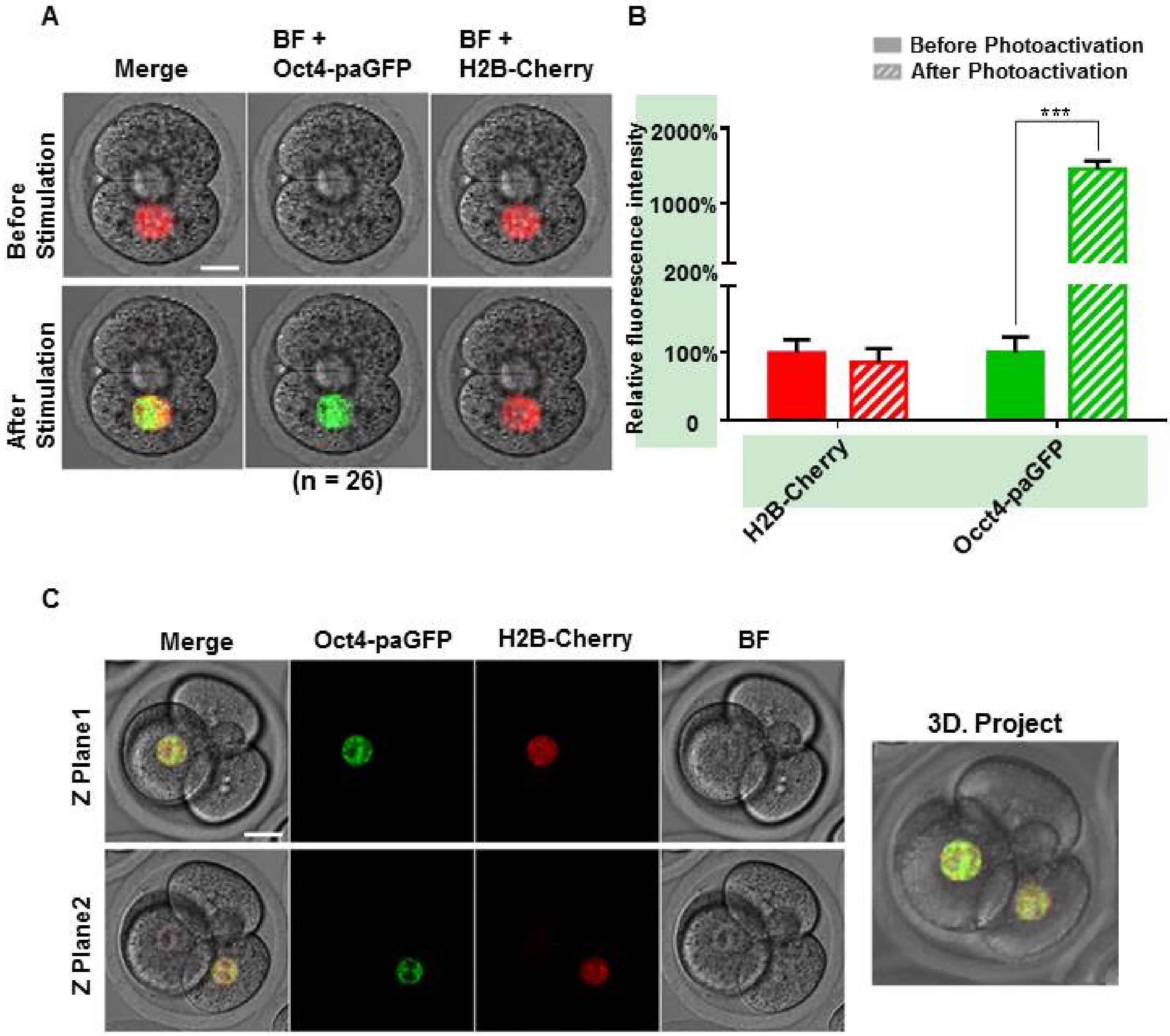
A
Merge
BF +
Oct4-paGFP
BF +
H2B-Cherry
Before
Stimulation
After
Stimulation
(n = 26)
B
Before Photoactivation
After Photoactivation
***
Relative fluorescence intensity
2000%
1000%
200%
100%
0
H2B-Cherry
Occt4-paGFP
C
Merge
Oct4-paGFP
H2B-Cherry
BF
Z Plane1
Z Plane2
3D. Project

42

Figure S4. Images of Oct4-paGFP fluorescence decay after photoactivation (FDAP) assay.

(A) A 2-cell stage embryo with Oct4-paGFP and H2B-mCherry expression before and after photoactivation**,** H2B-Cherry (Marker, red), Oct4-paGFP (green), 3D projections (max. proj.) of merged images are shown in the right panels. Scale bars, 20 μm.

(B) Relative fluorescence intensity of Oct4-paGFP and H2B-mCherry before and after photoactivation, the bar and whiskers indicate means and SD, ***$p < 0.001$.

(C) The same 4-cell stage embryo with Oct4-paGFP and H2B-mCherry expression in different focus planes. Scale bar, 20 μm.
