# Supplementary material for: Differential regulation of H3S10 phosphorylation, mitosis progression and cell fate by Aurora Kinase B and C in mouse preimplantation embryos
Source: Protein Cell. 2017 Apr 22;8(9):662–74. doi: 10.1007/s13238-017-0407-5 (PMC5563281; doi:10.1007/s13238-017-0407-5)
Supplement: Supplementary file 13 — Supplementary material 13 (PDF 66 kb) [file 13238_2017_407_MOESM13_ESM.pdf]

Supplementary Table 1. Primers for Q-PCR

Gene	Accession	Forward	Reverse	Tm.
Actb	NM_007393.5	GTGTGACGTTGACATCC GTAAAGAC	TTGCTGATCCACATCTGCT GGAAGG	60
AurkA	NM_001291185.1	CGGGTCCTGTGTCCTTC TAA	GTCTTCGGTCTTCTGCAAG G	60
AurkB	NM_011496.2	CCTGAAACATCCCAAC ATCC	CTCCCTGCAGACCTAACAG C	60
AurkC	NM_020572.2	TGGTTGATCTGTGGTGC ATT	CACAGAGCCTGGAGACCTT C	60
Oct4	NM_001252452.1	GGCTTCAGACTTCGCCT CC	AACCTGAGGTCCACAGTAT GC	60
Nanog	NM_001289828.1	ATGAAGTGCAAGCGGT GGCAGAAA	CCTGGTGGAGTCACAGAGT AGTTC	60
Klf4	NM_010637.3	ACAGGCGAGAAACCTT ACCACTGT	GCCTCTTCATGTGTAAGGC AAGGT	60
Nr5a2	NM_001159769.2	TCTCACACACAGAAGTC GCGTTCA	TGCAGGTTCTCCAGGTTCT TCACA	60
Prdm14	NM_001081209.2	CTCTTGATGCTTTTCGG ATGACT	GTGACAATTTGTACCAGGG CA	60
