# Supplementary material for: Differential regulation of H3S10 phosphorylation, mitosis progression and cell fate by Aurora Kinase B and C in mouse preimplantation embryos
Source: Protein Cell. 2017 Apr 22;8(9):662–74. doi: 10.1007/s13238-017-0407-5 (PMC5563281; doi:10.1007/s13238-017-0407-5)
Supplement: Supplementary file 14 — Supplementary material 14 (PDF 69 kb) [file 13238_2017_407_MOESM14_ESM.pdf]

Supplementary Movie Legends

Movie S1. Time lapse movies of Securin-mCherry degradation during 2 to 4-cell division in mouse preimplantation embryos. Chromosomes of injected blastomeres were labelled with H2B-GFP.

S1A: Representative control embryo.

S1B: Representative AurkB-OE embryo.

S1C: Representative AurkC-OE embryo.

S1D: Representative siAurkB embryo.

S1E: Representative siAurkC embryo.

Movie S2. Time lapse movie of AurkC-KD induced cytokinesis failure during 2 to 4-cell division in mouse preimplantation embryos. Chromosomes of injected blastomeres were labelled with H2B-GFP.

Movie S3. Time lapse confocal movies of Oct4-paGFP degradation in 4-cell mouse preimplantation embryos. Nucleus of injected blastomeres were labelled with H2B-mCherry.

S3A: Representative control embryo.

S3B: Representative AurkB-OE embryo.

S3C: Representative AurkC-OE embryo.

S3D: Representative siAurkB embryo.

S3E: Representative siAurkC embryo.
