# Supplementary material for: Differential regulation of H3S10 phosphorylation, mitosis progression and cell fate by Aurora Kinase B and C in mouse preimplantation embryos
Source: Protein Cell. 2017 Apr 22;8(9):662–74. doi: 10.1007/s13238-017-0407-5 (PMC5563281; doi:10.1007/s13238-017-0407-5)
Supplement: Supplementary file 15 — Supplementary material 15 (PDF 66 kb) [file 13238_2017_407_MOESM15_ESM.pdf]

Supplementary Table 2. Sequence of AurkB and AurkC siRNA.

Gene	Accession	siRNA	Forward	Reverse
AurkB	NM_011496.2	siAurkB1	AGAAGUUGGCUGAGAACAA	UUGUUCUCAGCCAACUUCU
		siAurkB2	CCAAGCUGCUCAAACAUAATT	AAUUAUGUUUGAGCAGCUUGG
AurkC	NM_020572.2	siAurkC1	GGGUGCUUCCUUGCUAGUU	AACUAGCAAGGAAGCACCC
		siAurkC2	CCAGGAAGCAUUUCACCAUTT	AAAUGGUGAAAUGCUUCCUGG
		siAurkC3	GGACAUCAAGCCAGAGAAU	AUUCUCUGGCUUGAUGUCC
		siNC	MISSION® siRNA Universal Negative Control #2, SIC002, SIGMA	
